# Bouncing Back! Counteracting Muscle Aging With Plyometric Muscle Loading

**DOI:** 10.3389/fphys.2019.00178

**Published:** 2019-03-05

**Authors:** Martino V. Franchi, Elena Monti, Austin Carter, Jonathan I. Quinlan, Philip J. J. Herrod, Neil D. Reeves, Marco V. Narici

**Affiliations:** ^1^Laboratory for Muscle Plasticity, Department of Orthopedics, Balgrist University Hospital, University of Zurich, Zurich, Switzerland; ^2^Sports Medicine Research Group, Department of Orthopedics, Balgrist University Hospital, University of Zurich, Zurich, Switzerland; ^3^Department of Biomedical Sciences, Institute of Physiology, University of Padua, Padua, Italy; ^4^MRC-ARUK Centre for Musculoskeletal Ageing, University of Nottingham, Derby, United Kingdom; ^5^School of Sport, Exercise and Rehabilitation Sciences, University of Birmingham, Birmingham, United Kingdom; ^6^School of Healthcare Science, Manchester Metropolitan University, Manchester, United Kingdom

**Keywords:** aging, sarcopenia, dynapenia, stretch-shortening cycle, muscle architecture, muscle power, muscle remodeling

## Abstract

The preservation of muscle power is crucial in aging for maintaining mobility and performing daily tasks. Resistance training involving high movement velocities represents a valid strategy to slow down the rate of sarcopenia, counteracting the loss of muscle mass and muscle power. Plyometric exercise may represent an effective training modality for increasing muscle power; however, its application in older populations has been sparingly investigated, as the high impact actions involved may reduce its feasibility for older individuals. By adopting a safer modality of plyometric training, we investigated if a 6-week plyometric training intervention could increase knee extensor muscle size, architecture, force and power in 14 young (YM, age = 25.4 ± 3.5 y; means ± SD) and nine older males (OM, age = 69.7 ± 3.4 y). Volunteers trained 3 times/week using a device similar to a leg press machine where the user was required to bounce against his body mass on a trampoline. Pre-to-post training changes in isometric maximum voluntary torque (MVT), leg extension power and vastus lateralis (VL) architecture were assessed. Muscle power increased in both groups (+27% OM -*P* < 0.001, 20% YM -*P* < 0.001), although the total external work performed during the training period was significantly lower for OM (i.e., ~-47%). Both groups showed significant increases in muscle thickness (MT) (+5.8 OM -*P* < 0.01 vs. +3.8% YM -*P* < 0.01), fascicle length (Lf) (+8% OM -*P* < 0.001 vs. +6% YM -*P* < 0.001), and pennation angle (PA) (+7.5% OM -*P* < 0.001 vs. +4.1% YM -*P* < 0.001). The current study shows that trampoline-based plyometric training is an effective intervention producing a rapid increase in muscle mass and power in both young and older individuals. The training modality used in this study seems to particularly benefit the older population, targeting the morphological and functional effects of sarcopenia in human muscle.

## Introduction

It has been estimated that 5–13% of people older than 60 years show a significantly increased rate of muscle mass loss (Morley et al., 2014). This prevalence escalates to 50% in people over 80 years indicating that physiological muscle loss in aging drastically accelerates between the age of 70 and 80 years (Morley et al., 2014). This progressive age-related decrease in muscle mass, associated with a loss of muscle strength, represents the current definition of sarcopenia (Cruz-Jentoft et al., 2010; Mitchell et al., 2012) a geriatric syndrome predisposing to poor outcomes such as mobility disorders, disability, poor quality of life, and increased risk of death (Cruz-Jentoft et al., 2010). Critically, the loss of muscle strength is far greater than that of muscle size, and at the age of 80 years it is four-fold greater (Moore, 2014). In today's population, with a proportion of elderly citizens exceeding that of young people, this is a real concern. Considerably more so since impaired muscle function has been shown to be a predictor of hospitalization, disability, and mortality (Roubenoff and Hughes, 2000; Metter et al., 2002; Delmonico et al., 2007; Cruz-Jentoft et al., 2010; Guadalupe-Grau et al., 2015).

Resistance exercise has widely been advocated among the best strategies for counteracting the decrease of skeletal muscle mass in older age; however, there is no consensus on the optimum form of exercise. In fact, the general opinion is that the frequency, intensity, volume, and/or mode of the optimal exercise warrant further investigation into how best prevent muscular decline in aging individuals. What is agreed, however, is that preservation of muscle power (the ability to accomplish muscular work per unit time) is fundamental for combating the decrease of physical performance associated with sarcopenia (Fielding et al., 2011; Quinlan et al., 2017). In this regard, the preservation of strength and velocity of movement in older adults is closely related to the ability to generate muscle power, which is fundamental for maintaining mobility and for the performance of simple daily tasks such as negotiating a flight of stairs, raising from a bed or chair or walking to the grocery store (Miszko et al., 2003; Reid and Fielding, 2012). Unfortunately, with aging, muscle power declines at a faster rate than muscle strength (Skelton et al., 1994; Izquierdo et al., 1999; Reid and Fielding, 2012; Alcazar et al., 2017; Cadore and Izquierdo, 2018). In fact, Evans (Evans, 2000) stated that a decrease in muscle power has more significant implications for risk of hip fracture, performance in daily tasks, and functional independence than a decrease in strength. Therefore, it seems imperative that maintenance of power through rapid force-generating exercises should be implemented in exercise programs for the elderly (Cadore et al., 2018).

In the last decades, plyometric exercise has become increasingly popular for increasing muscle power (Markovic and Mikulic, 2010). Plyometric training utilizes the stretch-shortening cycle (SSC), a term that describes movement tasks where the muscle-tendon unit is stretched and then shortened rapidly (Cardinale et al., 2011), and in which the elastic properties of a muscle are involved to generate maximal power production. The use of plyometrics within the older population has previously been advocated as a potential preventive measure against sarcopenia (Faulkner et al., 2007). A major problem that hampered the application of such programs is that plyometric exercises involving repeated cycles of fast deceleration followed almost immediately by rapid acceleration of the body in the opposite direction seem hard to perform for older individuals without risk of injury. To circumvent this problem, in the present study we used the Tramp-Trainer machine (FREI AG, Hinterzarten, Germany, EU) consisting of a trampoline attached to an inclined sledge, enabling the performance of repeated plyometric jumps while the subject is sitting on a chair with the back fully supported. With this device, exercise is performed seated and with a defined trajectory, which may reduce injury risk.

Hence the aim of the present study was to test the hypothesis that plyometric training, based on exercise against one own's body mass, would be effective for increasing muscle mass and power in older individuals.

## Materials and Methods

Fourteen young participants (height = 176.1 ± 6.3 cm, mass = 72.2 ± 13.8 kg, age = 25.4 ± 3.5 years (20-32 years range); data expressed as means ± SD) and 9 older volunteers (height = 172.1 ± 3.1 cm, mass = 79.1 ± 6.8 kg, age = 69.7 ± 3.4 years (65-76 years old range)) were recruited to undergo a 6-week plyometric training program. All participants were healthy, fully independent and recreationally active but not practicing regular vigorous physical activity and had not been engaged in any plyometric or strength training programs within the past 6 months. All participants were medically screened by means of a medical questionnaire to exclude sufferers of joint disease and metabolic, respiratory or cardiovascular impairments. All subjects provided written, informed consent. This study was carried out in accordance with the Declaration of Helsinki. The protocol was approved by The University of Nottingham Ethics Committee.

### Trampoline-Trainer Exercise

**Figure 1:**
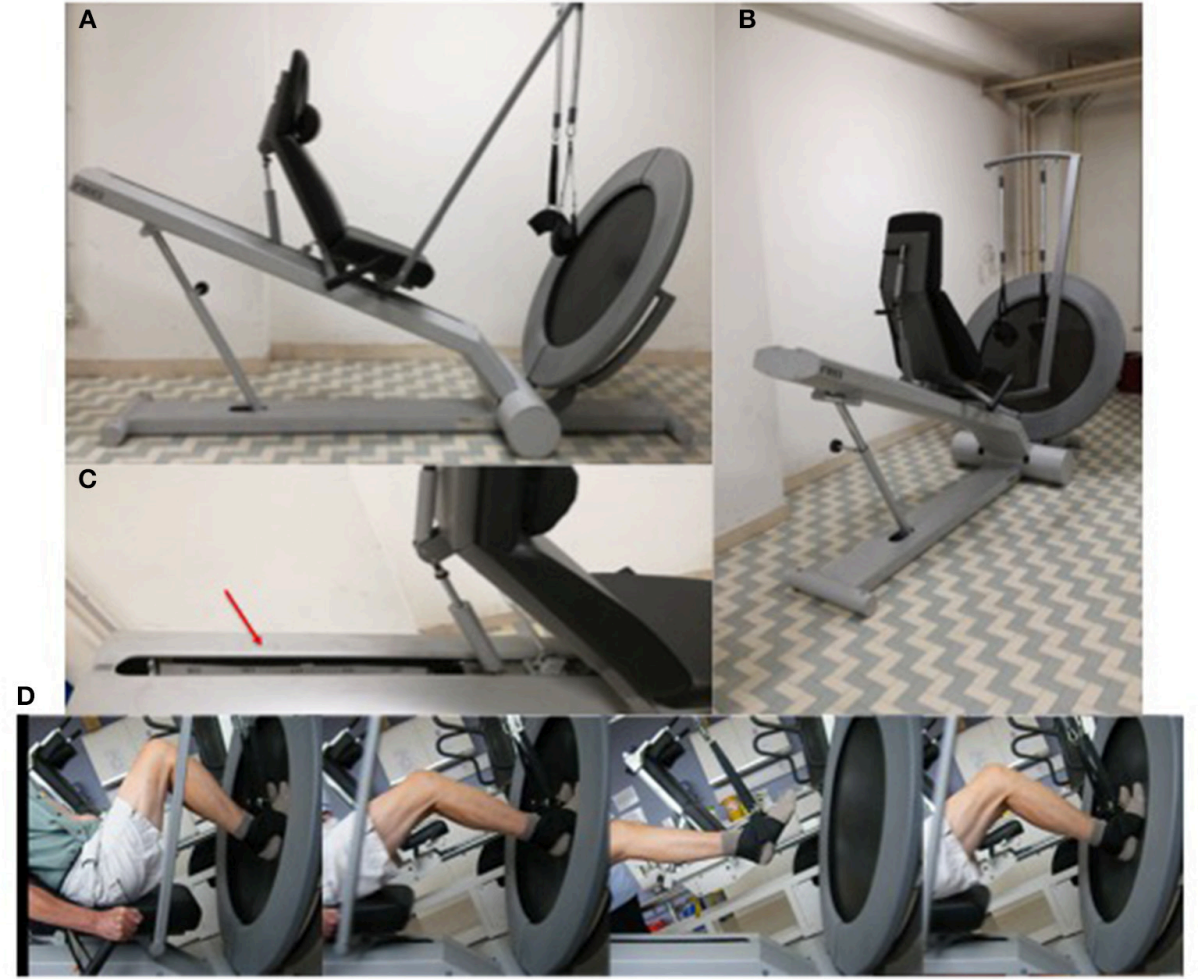
**(A,B)** Trampoline Trainer exercise device. **(C)** The red arrow points to the meter scale applied to the rail track of the TT device. **(D)** Representation of a bounce sequence performed by an elderly volunteer.

Training was performed on the “Tramp-Trainer” (TT) exercise machine (Figure 1) (FREI AG, Hinterzarten, Germany, EU). Briefly, the TT is a device similar to a leg press machine, except that the user is required to flex and extend the lower limbs (supported by two ankle braces connected to a spring, as shown in Figures 1A,B) against his/her own body weight on a trampoline, and thus on a compliant and elastic surface. The user is seated in a chair attached to a 1.5 m carriage with adjustable inclination, which allows the workload to be modified. In the current study, inclination was set to 22° (maximum incline) to elicit the greatest possible response to exercise. Essentially, the exercise on the Trampoline Trainer started from a semi-squat position with the knees flexed to a range between 90° and 80° (0° = anatomical zero/full leg extension) followed by a maximal push of the lower limb muscles (hip extensors, knee extensors and plantar flexors). The body and chair are then displaced along the rail during the jump, followed by landing on the elastic trampoline and immediate recoil as the subject bounces back; the entire cycle is then repeated for successive jumps (Figure 1D).

### Training Protocol

Training was performed 3 times per week for a period of 6 consecutive weeks. Training volume was based upon the guidelines of (Chu, 1998), stating that beginner and intermediate status athletes should not exceed 120 foot contacts per session when implementing a new plyometric training program. In the young group, training volume was therefore fixed at 4 sets x 30 repetitions for the first 4 weeks, followed by 5 sets × 30 repetitions for the final 2 weeks. In the elderly group, training volume was fixed at 3 sets × 30 repetitions for the first 4 weeks, followed by 4 sets × 30 repetitions for the final 2 weeks. From pilot data it became obvious that the initial increase in training volume after 2 weeks was too strenuous for the elderly group to manage, hence in both populations the training volume was increased after 4 weeks, rather than after 2 weeks.

Training load was matched across subjects by determining 30 Repetition Maximum value before the start of the program. This load equates to that which subjects could perform no more than 30 repetitions within a set without a decrease in bounce performance. A graduated scale marked in centimeters was fixed on the inside side of the rail-track (Figure 1C) of the Tramp Trainer to monitor bounce height during the exercise. Thus, 30-RM was first tested. Then, during the training protocol, subjects were instructed to bounce to a height that corresponded to their 30-RM, and if they fell 5 cm or more below their 30-RM height for up to 3 consecutive bounces, they were prompted to increase bounce height in order to maintain a constant training load.

As training progressed, loading increased progressively to maintain the training load at 30RM. The 30RM values were re-assessed every 7 days (before the start of the first session each new training week), thus new 30RM jump height values were identified. When volunteers reached the highest value of the meter scale attached to the carriage (i.e., the top of the carriage) for ~90% of the repetitions in a single training session, in order to increase the training load, a 15 kg weighted vest was provided from the successive session in order to further increase the training load. Successively, training load increase was provided by increasing the number of series from week 4 of the training period.

During a familiarization session on the TT, each participant practiced starting and landing in repeated jumps from a knee angle of ca. 90° measured with manual goniometer (full knee extension was taken as anatomical 0). During each training session a red mark was placed on the side of the inclined plane rail-track as a visual target corresponding to a knee flexion of ca. 90°.

### Isometric Maximum Voluntary Contraction Torque

Isometric muscle torque was measured using an isokinetic dynamometer (Cybex Norm, Cybex International Inc., NY, USA) at a joint angle of 70°, with full extension corresponding to 0°. After a brief warm-up, subjects performed two maximum voluntary contractions, which lasted for 4 s, with a rest period of 30 s between contractions. Subjects were provided with real time visual feedback of torque production during isometric contractions. The maximum isometric torque value (MVCT peak) was chosen for data analysis.

### Muscle Power Test

Leg extension power was assessed using the Nottingham Power Rig (Nottingham University, Nottingham, UK). The power rig provides a measure of peak power (W) generated by the lower limbs during a hip and knee extension movement (Bassey et al., 1992). The specifics of such device are described in detail by Bassey and Short (1990). Briefly, the device consist of a seat, and a lever (on which the feet are placed in order to exert force) which is connected to a flywheel by a chain. The leg extension movement is completed in 0.25–0.40 s (Bassey and Short, 1990). The resistance to the movement is minimal and remains nearly constant throughout the whole movement (Bassey and Short, 1990). During the testing sessions of the present study, subjects were asked to push as hard and as fast as possible (i.e., at their maximum velocity) on the raised foot plates through the full range of movement (5 repetitions). If subjects showed progressive improvements in peak power in the 5 repetitions, unlimited efforts were allowed until a plateau of performance was reached.

### Total External Mechanical Work

The average total external mechanical work (W_ext_, kj) performed throughout the study by both young and elderly group was calculated. Individual W_ext_ was calculated from body mass (plus 15 kg if subject were wearing the weighted vest), TT inclination, and average height reached during each bounce performed in each session (relative to the maximum height of the track/carriage).

For a single set of 30 bounces, W_ext_ was calculated as follows:

(1) $$\begin{array}{r} W_{ext}\left(kj\right)=\left[\left(BM+VM\right)xgx\left(\sin22\right)\right]x\left(1.265-h_{ex}\right)x30 \end{array}$$

where *BM* (kg) represents the subject body mass, *VM* (kg) the mass of the weighted vest, *g* is the gravitational acceleration (9.81 m^*^s^−2^) multiplied by the sine of the pre-set inclination of the track, 22°, which was kept constant throughout the whole study), *h*_*ex*_ (m) represents the average height reached by every bounce in the set (relative to the total length of the track, 1.265 m), and 30 is the number of bounces performed per set.

### Muscle Morphology and Architecture

**Figure 2:**
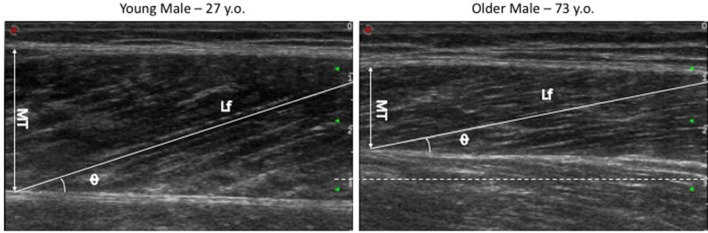
Vastus lateralis US images of Young vs. Old volunteers (acquired with same scale and US preset parameters). The dotted white line represents the 3 cm scale value, highlighting that images share the same starting point (0 cm) and that the representative image from the old volunteer shows a significantly smaller MT compared to the young participant.

Muscle architecture of the vastus lateralis muscle was measured *in vivo* using B-mode ultrasonography (Mylab70, Esaote, Genoa, Italy) (Figure 2). The measurements were acquired not more than 7 days before the start of the training period (in which the participants were asked to refrain from any occasional strength training session or strenuous exercise) and between 3 and 5 days after the last training session. Ultrasound images were obtained from the subjects' right leg when they were resting in a supine position. Vastus lateralis fascicle length (Lf), pennation angle (PA), and thickness (MT) were measured using a 10 cm, 10–15 MHz, linear-array probe, according to the method described in detail by Franchi et al. (2018b). Briefly, the probe was positioned over the belly of the vastus lateralis, carefully adjusted to the fascicle plane while the minimal pressure was applied (as described Franchi et al., 2017b). Ultrasound scan images were then analyzed using ImageJ image analysis software. Lf was determined through extrapolation of fibers and aponeuroses if a portion of the fascicle extended outside of the captured ultrasound image (as described Franchi et al., 2014, 2018a). PA was measured at the intersection between the fascicles and the deep aponeurosis. MT was measured as the perpendicular distance between the superficial and deep aponeuroses (Franchi et al., 2017b).

### EMG Evaluation During a Single Counter-Movement Jump (CMJ) Task

Electromyographic activity (EMG) was recorded from the vastus lateralis during a maximal vertical jump testing (CMJ). This design was adopted as CMJ is very similar to the movement performed on the training device and also because it represents a valid and well standardized performance test. Bipolar (20 mm inter-electrode distance) surface EMG recording was employed using two electrodes placed 94 ± 13.2 mm along a line from the superior lateral side of the patella, to the anterior superior iliac spine, starting from the patella (Rainoldi et al., 2004). The ground electrode was placed on the lateral side of the patella. Before positioning the electrodes, the skin was shaved, gently abrased using emery paper, and cleansed using alcohol swabs to reduce impedance below 5 KΩ. Raw EMG signal was acquired with a sampling frequency of 1000 Hz and processed with a multichannel analog-digital converter (EMG 100C, Biopac systems inc., Santa Barbara, CA, USA). The raw signal was then filtered using high- and low-pass filters set at 10 and 500 Hz, respectively, digitized, stored and analyzed by Acqknowledge software (Biopac systems inc., Santa Barbara, CA, USA). Root mean square (RMS) during a CMJ was calculated over a 200-ms time frame which corresponded to ±100 ms from the RMS peak, taking into account the electromechanical delay (EMD). This was set at 43.4 ms for the elderly group (Reeves et al., 2004), and 32.8 ms for the young group (de Boer et al., 2007). EMD is defined as the delay between the onset of electrical activity and torque. The onset of electrical activity was defined as an increase of 15 mV above baseline (Reeves et al., 2004).

### Rate of Perceived Exertion (RPE) and Muscle Soreness

Rate of perceived exertion (RPE) was monitored using the Borg Scale (Borg, 1998) at the end of each training sessions; participants were asked to visually indicate the level of general fatigue perceived. In addition, muscle soreness of the lower limbs was monitored using the soreness scale (Cook et al., 1997), before the start of every training and after each set (4 times/training) (immediate soreness after performing a set of 30 maximal bounces).

Visual supports representing each level of the scale (from 1 to 10 for both scales) were provided to help volunteers familiarizing with the scale itself and expressing the correct value of fatigue or soreness when asked.

### Blood Pressure and Heart Rate

Because of the novelty of such training modality, for safety reasons, only the OM group was monitored during training by a medical doctor who checked, before and after each set, the heart rate (HR) and diastolic and systolic blood pressure (BP). Parameters were recorded in a “health monitor diary” and average values were calculated for week 1 and week 6 of the training period.

### Statistical Analysis

Differences in the functional, morphological, and architectural muscle components of the study between groups (young vs elderly) and time (baseline vs post training) were investigated using a two-way repeated measures ANOVA. Bonferroni's multiple comparisons test was used to identify significance within groups from baseline to 6 weeks (post training). Differences between groups in the total external mechanical work and pre-to-post training increase percentage in MT and Power (i.e., also normalized by work) were evaluated by an unpaired Student's *T*-test. GraphPad Prism software (version 7.0; GraphPad software Inc. San Diego, CA) was used to perform all statistical and *post hoc* analysis.

## Results

The average values for functional and morphological adaptations of each group at each time point are presented in Table 1 (means ± S.D).

**Table 1:** Pre-to-post values for muscle functional and morphological features.

	**Young males**		**Older males**	
	**Baseline**	**6 Weeks**	**Baseline**	**6 Weeks**
MVC (Nm)	246.15 ± 50.69	260.35 ± 50.16	204.75 ± 43.98	221.03 ± 42.87
Power (W)	423.83 ± 136.33	506.08 ± 153.23^***^	327.26 ± 82.08	408.21 ± 107.97^***^
CMJ EMG (mV)	0.23 ± 0.10	0.30 ± 0.13^**^	0.15 ± 0.06	0.21 ± 0.07^*^
VL MT (cm)	2.44 ± 0.34	2.54 ± 0.35^**^	1.96 ± 0.32^∧^	2.09 ± 0.33^**^^,^^∧^
VL PA (deg)	16.35 ± 1.36	17.03 ± 1.63^***^	14.48 ± 2.32^∧^	15.51 ± 2.08^***^
VL Lf (cm)	7.66 ± 0.90	8.12 ± 0.91^***^	7.51 ± 0.41	8.15 ± 0.39^***^
RPE	5.18 ± 1.53	3.33 ± 1.81^**^	4.45 ± 1.91	3.65 ± 1.62
Soreness scale	3.33 ± 1.84	1.49 ± 1.82^**^	1.85 ± 2.01	1.21 ± 1.73

Baseline vs. 6 Weeks differences:

* *P < 0.05*;

** *P < 0.01*;

*** *P < 0.001*.

Between groups differences (at the same time-point):

∧ *P < 0.05*,

*^∧∧^ P < 0.01*.

### Isometric Maximum Voluntary Contraction Torque

The average group values for Isometric MVCT were greater after 6 week of plyometric training compared to baseline in both young (246.2 ± 50.7 Nm vs. 260.4 ± 50.2 Nm, *p* = 0.09) and older males (204.8 ± 44 Nm vs. 221.9 ± 40.4 Nm, *p* = 0.13) (increase percentage values = 6.8% ± 12.6 -YM vs. 8.6% ± 9.9% -OM). Due to the high individual variance of such responses, such increases in MVCT were not significant, neither between time points nor between groups.

### Maximum Leg Extension Power

Muscle Power (leg extension power) increased significantly after the 6-weeks training period compared to baseline in both young and older male groups (423.9 ± 136.4 W vs. 506.1 ± 153.2 W, *P* < 0.001, and 327.3 ± 82.1 W vs. 408.2 ± 108 W, *P* < 0.001, respectively) (increase percentage values = 20% ± 11, *P* < 0.001 -YM vs. 27% ± 11, *P* < 0.001 -OM), without significant differences observed between groups.

### Muscle Architecture

Vastus lateralis fascicle length (Lf) presented a significant increase in both groups after training (YM = 7.66 ± 0.9 cm vs. 8.12 ± 0.9 cm, *P* < 0.001, and OM = 7.51 ± 0.4 cm vs. 8.15 ± 0.4 cm, *P* < 0.001) (increase percentage values = 6.1% ± 3.1%, *P* < 0.001 -YM vs. 8% ± 2.5%, *P* < 0.001 -OM) without significant differences observed between groups. Baseline values were not significantly different between groups.

Pennation angle (PA) presented significant changes in both groups (YM = 16.4 ± 1.4° vs. 17 ± 1.6°, *P* < 0.01, and OM = 14.5 ± 2.3° vs. 15.5 ± 2.2°, *P* < 0.001) (increase percentage values = 4.1% ± 3.4%, *P* < 0.01 -YM vs. 7.5% ± 3.2, *P* < 0.001 -OM). The increase was significantly greater in OM compared to YM (*P* < 0.05). Baseline values were significantly different between groups (P < 0.05); however, this between-groups difference was lost at the 6-week time point.

Muscle thickness (MT) increased significantly pre-to-post training in both groups (YM = 2.44 ± 0.3 cm vs. 2.54 ± 0.3 cm, *P* < 0.01, and OM = 1.96 ± 0.3 cm vs. 2.09 ± 0.3 cm, *P* < 0.01) (increase percentage values = 3.8% ± 3.4%, *P* < 0.01 -YM vs. 5.8% ± 5%, *P* < 0.01 -OM) without significant differences observed between groups. Baseline absolute values were significantly different between groups (*P* < 0.05) and so they remained at the 6-week time point (*P* < 0.05).

### EMG RMS During a CMJ task

EMG RMS evaluated during a maximal CMJ presented a significant in both groups after the training program. The changes were similar in both young and older groups, as YM showed a 42% increase (*P* < 0.01) and the OM showed an increase of 46% (*P* < 0.05) compared to baseline values.

### Total External Mechanical Work Performed

**Figure 3:**
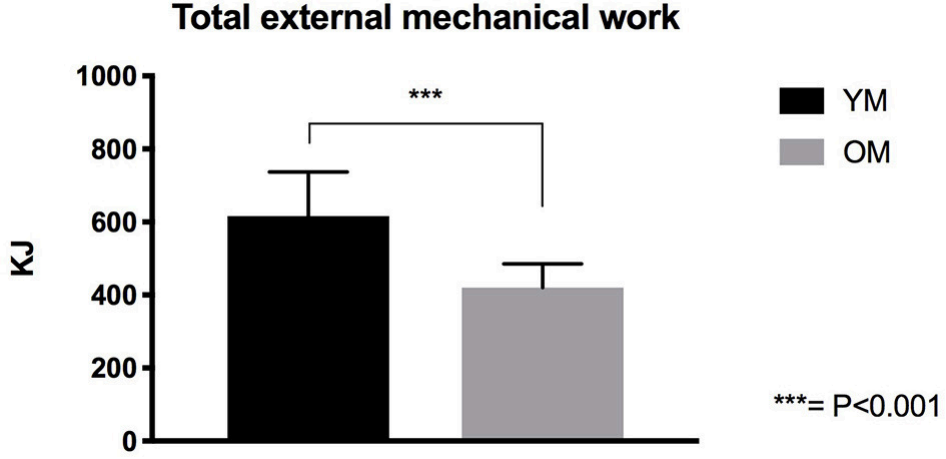
Total External mechanical work performed by YM and OM groups.

Total external mechanical work performed in the 6-week training period was found significantly different (*P* < 0.001) between YM and OM groups. The average total work performed by the YM was 616.1 ± 121 kJ while that performed by the OM was 420 ± 65.2 kJ. Thus, over the 6-week training period, YM performed 46.6% more work than the OM group (Figure 3).

### Functional and Morphological Adaptations Expressed by Total External Mechanical Work Performed

**Figure 4:**
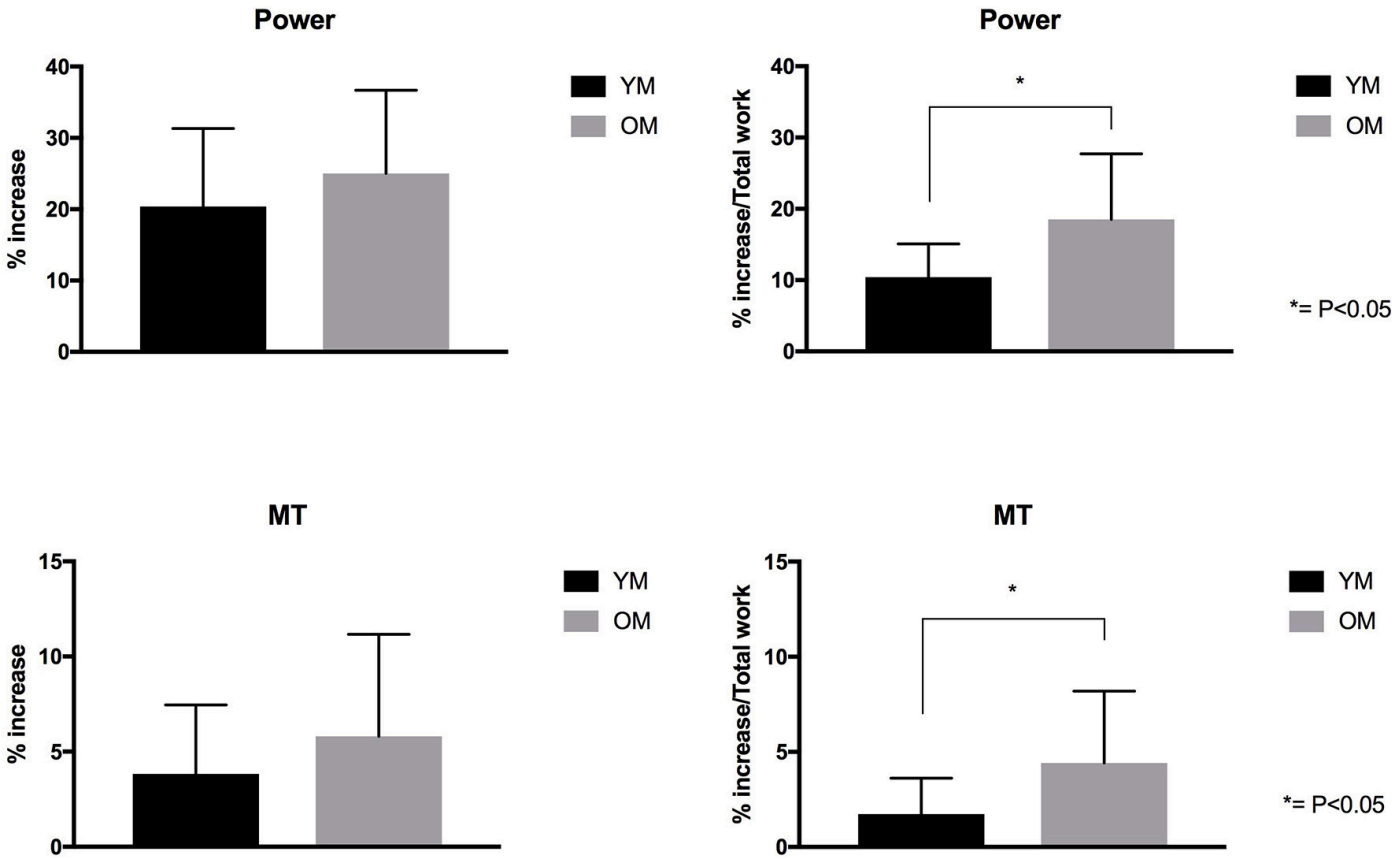
Power and MT% increase for YM and OM groups. On the right side of the figure, Power and MT expressed by Total External Mechanical Work performed.

When muscle power and MT percentage increase values were normalized by the total external mechanical work performed, a statistically significant difference was observed between YM and OM (power = 10.4% ± 4.6% vs. 18.5% ± 9.1%, respectively – *P* < 0.05; MT = 1.7% ± 1.8% vs. 4.4% ± 3.7%, respectively – *P* < 0.05). MVC did not clearly changealso when normalized for total external mechanical load (Figure 4).

### RPE and Soreness Scale

RPE and soreness scale values did not differ between YM and OM groups. However, both RPE and soreness scale values showed a significant decrement in the YM group from week 1 to week 6 time points (*P* < 0.01) (Table 1).

### Monitored Cardiovascular Parameters (Older Male Group Only)

In the 9 Older Males, heart rate (HR) and diastolic and systolic blood pressure (BP) were monitored before and after the training session on week 1 and week 6 of the training period. Pre and post-exercise session HR did not significantly change from week1 to week6 time points (pre-exercise session = 67.8 ± 33.2 vs. 67.7 ± 38 bpm, respectively; post-exercise session = 128.7 ± 17.4 vs. 126.1 ± 16 bpm, respectively). No significant differences were observed for pre-exercise session systolic and diastolic BP between week 1 and week 6 time points (140.4 ± 3.6/84.3 ± 10 mmHg vs. 136.5 ± 11.9/83.1 ± 9.6 mmHg, respectively). A strong trend (*P* = 0.06) was observed for significant reduction of post-exercise session systolic BP from week1 to week 6, whereas diastolic BP did not show any statistically significant differences with time (199.5 ± 18.7/95.3 ± 14.1 mmHg vs. 179.1 ± 24.4/89.4 ± 11.4 mmHg, respectively).

## Discussion

In this proof-of-concept study, we investigated whether plyometric exercise could be used as an efficient strategy to counteract sarcopenia in the elderly, as this may be expected to positively impact on mobility and quality of life (Evans, 2000). Accordingly, we investigated skeletal muscle morphological and functional adaptations to a plyometric training intervention in older and young individuals. The main findings show that plyometric exercise produces significant and rapid improvements in muscle power and size in both young and older participants. Knee extensor muscle power (measured through a maximal velocity knee extension on the Nottingham Power Rig device) increased by 27% in OM and 20% in YM after just 6 weeks of training. This observation seems extremely noteworthy as it shows that a substantial improvement of muscle power may be achieved in less than 2 months of resistive training. Interestingly, expressing this gain as daily rate, this equates to 0.64%/day for the OM group. This is identical to the percent daily gain which may be estimated from the study of Izquierdo et al. (2001), reporting a 36% increase in leg extensors muscle power after 8 weeks of heavy resistance training in 64-year old men, equating to a daily gain of 0.64%/day. Hence plyometric training seems to produce very similar gains in muscle power as those achieved by heavy resistance training over a period of 6–8 weeks of training.

Since mechanical power is the product of force and velocity, an increase in power output may be the result of an increase of muscle force, velocity or both. As the results of this study show, maximum isometric torque did not significantly change after the training in either OM nor YM, however fascicle length did in both OM (+8%) and YM (+6%) participants. Theoretically, assuming that the increase in fascicle length is reflective of an increase in fiber length due to an increase in sarcomere number (Williams, 1990), and assuming an optimum sarcomere length for the VL muscle of 2.73 μm (0.000273 cm) (Walker and Schrodt, 1974), then the observed fascicle length of 8.15 cm after training and 7.51 cm before training for OM and 8.12 cm after training and 7.66 cm before training for YM result in an estimated increase in sarcomere number of 2344 (8.15/0.000273 cm minus 7.51/0.000273 cm) for the OM and of 1685 (8.12/0.000273 minus 7.66 /0.000273) for the YM. We advise the reader to interpret this simplified mathematical calculation with caution, as recent work in animal (Moo et al., 2016, 2017) and human muscle (Lichtwark et al., 2018) has showed that sarcomere lengths are non-uniform within distinct muscle regions (Moo et al., 2016; Lichtwark et al., 2018) and even more inhomogeneous upon contraction (Moo et al., 2017). Therefore, this could represent a limitation of our mathematical simplification. Nonetheless, since the muscle maximum shortening velocity has been shown to be proportional to the number of sarcomere in series (Bodine et al., 1982), the estimated increase in sarcomere number may have contributed to the observed changes in muscle power. However, this contribution (8% in OM and 6% in YM) clearly does not fully account for the increase in muscle power (27% in OM and 20% in YM). Nonetheless, as our findings show, the increase in muscle power was accompanied by a significant increase in EMG activity (RMS) of the VL muscle (42% in YM and 46% in OM during CMJ). This finding suggests that improved muscle recruitment may have contributed to the increase in muscle power induced by the plyometric intervention of this study.

While a small but significant increase in MT in both OM (5.8%) and YM (3.8%) was observed, the increase in muscle strength (8.6% in OM and 6.8% in YM) did not reach statistical significance despite the presence of muscle hypertrophy. The increase in MT predicted a proportionate increase in muscle strength. It seems likely that the lack of torque increase reflected the type of resistive training used in this study, which was focused on muscle power rather than muscle strength, as gains in muscle strength have been shown to be training-specific (Tillin et al., 2012). In addition, the lack of significant changes observed for MVCT could reflect the non-matching movement specificity between the isometric MVCT and the TT training device.

A key outcome of the study is that, while both groups presented similar increases in MT and muscle power, OM performed significantly less total amount of external mechanical work compared to YM (~-47%) over the 6-week training period. Thus, by normalizing MT and power percentage increase values by the total work performed, such changes were markedly greater in the older population (Figure 4). The observation that OM achieved very similar hypertrophic responses to those in YM but while performing fewer repetitions/sets per week, with no use of the weighted jacket (i.e., no supplemental overload than the body weight), and consequently while performing less total external mechanical work compared to YM, is indeed noteworthy. This observation could indeed suggest that this training modality may result optimal for an elderly population. In addition, the increase in MT, expressed for the external work performed, presents a significant discrepancy of ~2.7% between age groups in favor to the elderly participants. However, when expressing the increase in muscle power for the total external work performed, the OM group showed a significantly greater capacity for augmenting in power (~8.1% discrepancy between groups) in the same time-frame. In addition, these responses were achieved after just 6 weeks of plyometric-based training, which represents a rather efficient time frame in rehabilitation and clinical settings. Previous research has shown that decreased muscle mass is associated with an increased risk of hip fracture (Cummings et al., 1995; Turner et al., 2004), and that, concomitantly, a marked reduction in power production represents a major risk factor for hip fracture among the elderly (Phillips et al., 1998). This has particularly important implications for sarcopenic populations, in which a decline of power has been observed faster than declines in isometric strength (Izquierdo et al., 1999) and knee extensor strength (Skelton et al., 1994). Leg muscle power has indeed been shown to be more important for performing daily activities than strength in frail elderly people (Bassey et al., 1992). The increase in power seen following plyometric exercise is thus likely to have positive implications for improving daily activities such as stair climbing, time to rise from a chair/toilet, and walking around the home. Bassey et al. (1992) showed that older men and women who performed these tasks with assistive aids had 42–54% less leg extensor power compared to those who performed the tasks without aids. Thus, the present findings suggest that this novel plyometric protocol may represent a time-efficient strategy to counteract sarcopenia in the aging population.

When considering skeletal muscle performance and aging, changes in the structure of muscular system undoubtedly acquire an important role, as architectural remodeling of skeletal muscle can influence its functional properties (Narici and Maganaris, 2007). In the present study, both YM and OM groups presented significant changes in muscle architecture (i.e., MT, PA and Lf). In the elderly group, the aforementioned increase in MT (5.8%) and in fascicle length (8%) was accompanied by increases in pennation angle (7.5%). These architectural adaptations are evidence of a hypertrophic response (i.e., increase in MT) and could reflect distinct strategies of new contractile material deposition (Franchi et al., 2017a), since an increase in PA may reflect the addition of sarcomeres in parallel (Narici et al., 2016) and an increase in Lf is indicative of a potential addition of sarcomere in series (Franchi et al., 2014, 2016). The YM group also presented significant changes in MT (3.8%), Lf (6.1%), and PA (4.1%, significantly less than the increase in OM); however, compared to the OM group, slightly weaker architectural responses to this specific training modality were observed in YM. These results warrant some considerations.

The significant different increase in PA between age groups suggests that the intensity of the training stimulus may not have been sufficiently high in the YM to elicit a greater response, especially considering that the young participants were not presenting any signs of sarcopenia to begin with, and therefore not as sensitive as the elderly to the training intervention (Komi, 2003). The increase in fascicle length following plyometric training seems to be in line with the work of Blazevich et al. (2003). The observation that the both the YM and OM group showed a similar strong increase in Lf may suggest that the eccentric phase of the stretch-shortening cycle have been the main driver of hypertrophic responses (i.e., the adaptations reflect the predominant fascicle behavior seen during the training). Theoretically, during the SSC the muscle is firstly stretched (deceleration phase–eccentric component) before it rapidly contracts concentrically (acceleration phase) (Bosco et al., 1981). However, it is still debated if the muscle undergoes a full eccentric phase during plyometric actions, as some work suggested that the tendon and the series elastic components are mostly lengthened, while the muscle fascicles behave “quasi-isometrically” (Finni et al., 2003; Ishikawa and Komi, 2004). Although there is evidence of significant (Ishikawa et al., 2006) (or a tendency for – Hirayama et al., 2017) fascicle lengthening when using high load, this mechanical stretch applied onto the fascicles may not be comparable to that observed in pure eccentric contractions (Franchi et al., 2014). Nevertheless, it has been previously shown that isometric contractions at longer muscle lengths (~90°) could lead to an increase in Lf (Noorkõiv et al., 2014). Lastly, the aponeuroses may have contributed to such adaptations in Lf, as they would have undergone longitudinal and transversal stretch during the SSC task, consequently resulting in significant stress applied onto the inserting fascicle (Raiteri, 2018). These events would in turn potentially trigger mechanotransductor signaling pathways, which have been recently associated to changes in muscle architecture (Franchi et al., 2018a). Taken together, all the aforementioned factors may play a determinant role in the remodeling of skeletal muscle following plyometric training in young and athletic populations.

Notably, these morphological changes were achieved in a relative short training period. Early muscle architectural remodeling has been previously documented in response to conventional, isokinetic and isoinertial resistance training (3–6 weeks duration) in young and older population (Blazevich et al., 2003, 2007; Seynnes et al., 2007; Franchi et al., 2015, 2018a; Brook et al., 2016) but they were not yet reported in response to plyometric training modalities, especially in older and/or sarcopenic men.

It is worth noting that the use if plyometric training for improving muscle mass, strength, power and endurance in old age has been previously advocated by Professor John Faulkner and colleagues (Faulkner et al., 2007), and our study seems to confirm the suitability of this type of exercise form improving muscle mass and power in old age. Up to now, only one study investigated the neuromuscular adaptations to a 12-weeks plyometric program in older adults (Piirainen et al., 2014). Piirainen and colleagues compared a plyometric training modality to a more classical pneumatic resistance training in older adults (60–70 years old). Plyometric training was performed on a custom-built sledge ergometer very similar to the TT device used in this study, with the only difference that the platform surface was hard and not compliant (Piirainen et al., 2014). Whereas knee extensor MVC significantly increased after just 4 weeks of pneumatic training, significant strength increase was only observed after 12 weeks in the plyometric group: this is in agreement with the lack of significant changes in MVC of the present study, that is MVC changes probably reach significance after 6 weeks of training.

A note of caution, however, ought to be expressed on the use of plyometric training. As it involves of repeated stretch -shortening contractions, the training program should be designed by certified exercise professionals in order to minimize the risk of “contraction-induced injury” to participants. Although in this study we did not measure muscle damage markers such as creatin kinase, significant muscle damage may be excluded as neither our older or younger participants reported an increase in the muscle soreness scale or any adverse effect after each exercise session.

## Conclusion

The current study showed that plyometric exercise is an effective tool in counteracting the morphological and functional effects of sarcopenia in human muscle in a training period of only 6 weeks. Although the total amount of external mechanical work was ~47% lower in the elderly group, both young and older individual achieved similar increases in muscle size and power through plyometric exercise. Thus, we have successfully demonstrated that in a group of elderly subjects, plyometric exercise can increase muscle power in a time-efficient manner. This may have significant implications for reducing risks of hip fractures, improving ability to perform daily tasks, and improving functional independence and to maintain quality of life in elderly individuals.

## Data Availability

The datasets generated for this study are available on request to the corresponding author.

## Author Contributions

MF, NR, and MN conceived the study. MF, MN, NR, and AC contributed to the study design assembly. MF, EM, AC, JQ, and PH perform the study and the data collection. MF, EM, and AC performed the data analysis. MF, AC, EM, and MN contributed to the data interpretation. MF, AC, EM, and MN drafted the manuscript; all authors approved the final version of the manuscript.

### Conflict of Interest Statement

The authors declare that the research was conducted in the absence of any commercial or financial relationships that could be construed as a potential conflict of interest.
